# Editorial: Genomics Research on Non-model Plant Pathogens: Delivering Novel Insights into Rust Fungus Biology

**DOI:** 10.3389/fpls.2016.00216

**Published:** 2016-02-23

**Authors:** Guus Bakkeren, David. L. Joly, Sébastien Duplessis

**Affiliations:** ^1^Agriculture and Agri-Food Canada, Summerland Research and Development CentreSummerland, BC, Canada; ^2^Department of Biology, Université de MonctonMoncton, NB, Canada; ^3^INRA, UMR 1136 Interactions Arbres/Microorganismes INRA/Université de Lorraine, Centre INRA Nancy LorraineChampenoux, France; ^4^Faculté des Sciences et Technologies, Université de Lorraine, UMR 1136 Interactions Arbres/Microorganismes Université de Lorraine/INRAVandoeuvre-lès-Nancy, France

**Keywords:** rust fungi, basidiomycota, obligate biotrophy, genomics, resequencing, genetic variation, transcriptomics, effectors

The diversity among rust fungi is simply astounding: over 7000 species that evolved to colonize niches all over the plant kingdom. This diversification likely involved major host jumps, especially considering that the life cycles of heteroecious rusts, such as the cereal rusts, involve sexual and asexual stages that take place on completely unrelated host plants (Savile, 1976; McTaggart et al., 2015), but also co-evolution in diverse natural settings, establishing equilibrium (e.g., Thrall et al., 2012). These interactions resulted in complex life styles including for many rusts, the production of up to five different spore types, each requiring very different developmental programs and hence gene expression.

Unfortunately, despite the intriguing biology of these fascinating pathogens, many rust fungi have gained notoriety because of the fact that some of their hosts were selected as food sources by humans leading to their extensive cultivation, and more recently their monocultures over large areas. Upsetting the balance, the rust fungi took the occasion, having a ball.

Because of their importance, plenty of research has been done on rust fungi describing life cycles, host range, and infection processes. Driven by the need for resistant crop cultivars, the genetics of race-cultivar interactions and fungal race identification pioneered in the 1940's, became the staple for breeding programs worldwide. However, because of their strict biotrophic life styles and recalcitrance to genetic transformation, molecular genetic research on rust fungi remains difficult. The advance of genomics has really impacted research on rust fungi, demonstrated by the expansion of labs embarking on and receiving funding for molecular work over the last 5 years. With this in mind, we invited submissions for this *Frontiers in Plant Science Research Topic* and present here 14 papers.

Recent studies have revealed that genome sizes vary widely among the rust fungi but are on average much larger than other basidiomycete genomes: 89 Mbp for *Puccinia graminis* f. sp. *tritici* and 101 Mbp for *Melampsora larici-populina* and harboring at least 45% repeat and transposable elements (Duplessis et al., 2011). Here we present papers indicating that this may generally be true. A high quality assembly of the *Melampsora lini* genome indicated a genome size of 189 Mbp with at least 45% harboring repetitive elements, primarily retrotransposons (Nemri et al.). A larger size is expected from the initial assembly of a draft genome of *Uromyces fabae* which here is estimated at 329 Mbp (Link et al.). Several groups have attempted to sequence the genome of the destructive Asian soybean pathogen *Phakopsora pachyrhizi*. It became clear early on that its genome was massive, possibly above 850 Mbp but here, a new report estimated it to be in the 500 Mbp-range (Loehrer et al.). To better prepare for genome sequencing projects, genome size estimates thus become important. Here, Tavares et al. employ nuclear fluorescence and flow cytometry to present rough estimates of the genome sizes of a wide variety of rust fungi, contrasting the sizes of the previously reported genomes. It is widely believed that active transposable elements contribute to and are responsible for these expanded, over-sized genomes since the gene space, and number of gene models predicted (and confirmed by RNAseq data in many cases) is roughly similar for all of them (Spanu, 2012). Indeed, it is believed that polyploidy and/or the activity of transposable elements could be important diversity-creating factors in such mostly asexual species, being the main mechanisms driving genome expansions (Ramos et al., 2015). The coffee rust fungus *Hemileia vastatrix* is among those exhibiting a particularly large genome and a striking richness in repeated elements, making it difficult to assemble genome reads. A draft genome of 333 Mb was generated through a hybrid assembly of eight different isolates and helped in the identification of more than 14,000 putative genes (Cristancho et al.). Performing genetic studies in the laboratory with heteroecious rust fungi is complex as it requires two hosts to generate the various spore stages for crossing. A major breakthrough is reported here on a successful self-cross of the reference genome *M. larici-populina* isolate and the benefit of resequencing offspring in order to identify recombination breaks in its genome (Pernaci et al.). This also allowed corrections to the initial (dikaryotic) genome assembly, generating a framework for the construction of a future genetic map.

Two transcriptomic studies of specific stages of plant-rust interactions (early infection and telia development) illustrate the importance of expression profiling in gaining an understanding of rust biology. Whereas most recent rust transcriptomic studies focus on time-course infections of the telial host or on isolated haustoria collected from such infected host tissues (see Duplessis et al., 2014), other stages have been less investigated. Here transcript profiles of the coffee rust fungus at early stages of the infection (i.e., germinating urediniospores and appressoria) were compared to previously reported expression profiles *in planta* highlighting candidate effector genes expressed only during biotrophic growth in the plant and also signaling pathways and metabolic shifts that most likely condition the success of infection (Talhinhas et al.). Teliospores are survival structures produced on decaying telial host tissues and molecular mechanisms responsible for this process are mostly unknown. Here, Hacquard et al. report on transcriptome profiling in early *M. larici-populina* telia sampled in autumn, revealing adaptation to the drastic change in environmental conditions and the tight coordination of karyogamy and meiotic processes. Comparing these profiles with poplar infection identified genes encoding small secreted proteins that most likely do not play a role in the biotrophic phase.

Available genome sequences have accelerated the discovery of candidate effectors, which can support the design of disease management and resistance breeding strategies (Vleeshouwers and Oliver, 2014). Because of their importance, a mini review on effector proteins of rust fungi has been included in this issue (Petre et al.). Predicting candidate effectors in fungal pathogen genomes has been difficult due to a lack of signature sequence motifs, resulting in too many candidates for functional validation. Here Nemri et al. established a bioinformatic pipeline that prioritizes effector candidates by integrating multiple lines of evidence, taking advantage of the knowledge acquired from flax rust Avr proteins and other known rust effectors. Pendleton et al. investigated gene gain and loss in rust fungi and found gene family losses and contractions, and numerous lineage-specific duplications, probably accounting for their large proteome size. As described above, their highly plastic genomes probably played a major role in this process. As found in *M. lini* (Nemri et al.), such lineage-specific families could be enriched in Avr proteins targeted by host intracellular immune receptors (i.e., R proteins). Working on *P. graminis* f. sp. *tritici*, Sperschneider et al. employed another similar pipeline based on taxonomic information, expression data and evolutionary signatures of diversifying selection. Among the 42 effector candidates identified as part of pathogen associated gene families up-regulated during infection and rapidly evolving, was one that was previously shown to trigger genotype specific defense responses in wheat (Upadhyaya et al., 2014). Surprisingly, most of those candidates lacked features frequently associated with fungal effectors such as small size and high cysteine content, reinforcing the need for unbiased predictors.

A lack of identifiable homologs or clustering in families could result in unidentified effector candidates. Comparative genomic and transcriptomic approaches can circumvent this bias by correlating mutations within candidate effector genes with virulence phenotypes. By sequencing the transcriptome of six wheat leaf rust races and focusing on genes encoding secreted proteins during infection, Bruce et al. found amino acid changes in 15 *P. triticina* effector genes that correlated with virulence shifts. Similarly, in *M. larici-populina*, correlations between non-synonymous mutations and avirulence phenotypes were uncovered using genomic resequencing data from multiple isolates (Persoons et al.). A similar study in *P. graminis* f. sp. *tritici* (Upadhyaya et al.) compared amino acid changes between a given isolate and field-derived mutants that have gained virulence toward certain *R* genes. Because of the close genetic relationship between these isolates, a relatively low number of SNPs were identified, reducing the number of candidate genes requiring experimental validation as potential molecular determinants of the observed recognition specificities.

Rust fungi are among the most devastating plant pathogens and a serious threat to major domesticated crops (Dean et al., 2012). Though their obligate biotrophic growth habit and complex lifestyles make these fungi difficult to study with most standard genetic and molecular tools and techniques, we show here in this research topic that the genomics revolution that we are currently experiencing leads to new opportunities to dissect the biology of these fascinating fungi. These studies represent a few examples illustrating that these novel genomic approaches can deliver on promises made, including anticipated progress toward crop protection.

## Author contributions

All authors listed, have made substantial, direct and intellectual contribution to the work, and approved it for publication.

### Conflict of interest statement

The authors declare that the research was conducted in the absence of any commercial or financial relationships that could be construed as a potential conflict of interest.
